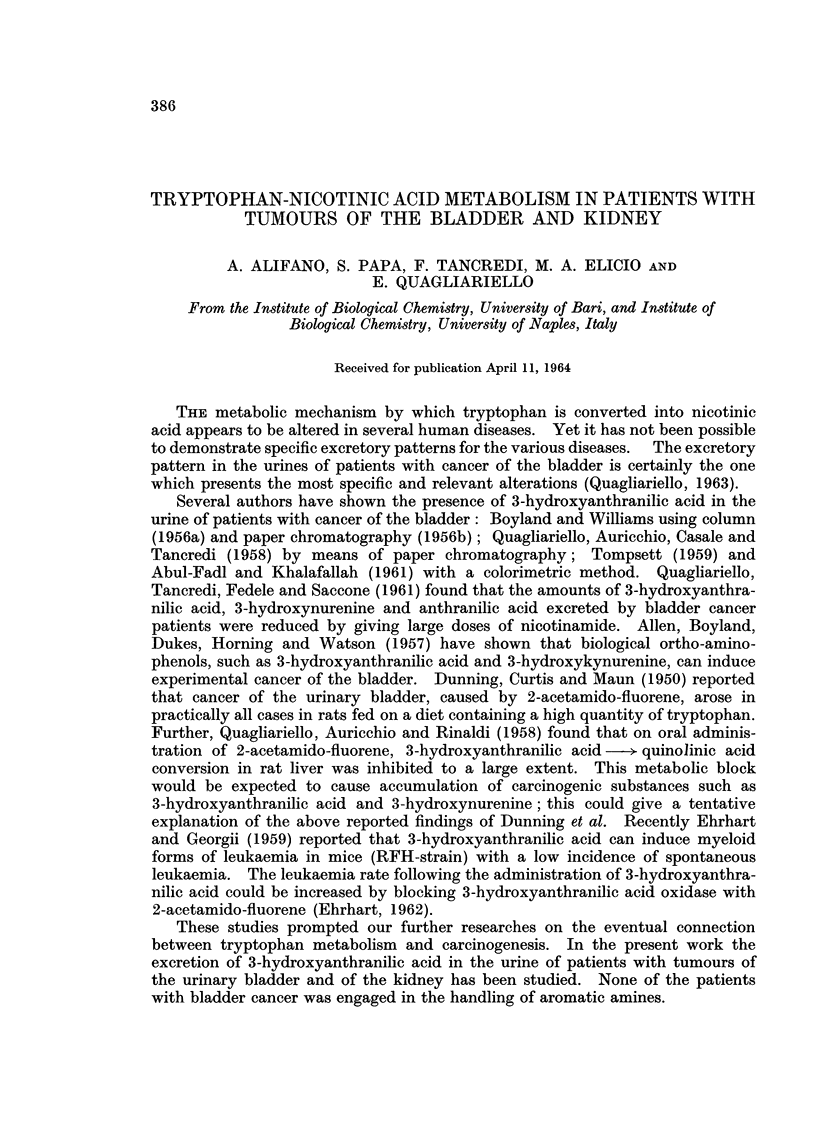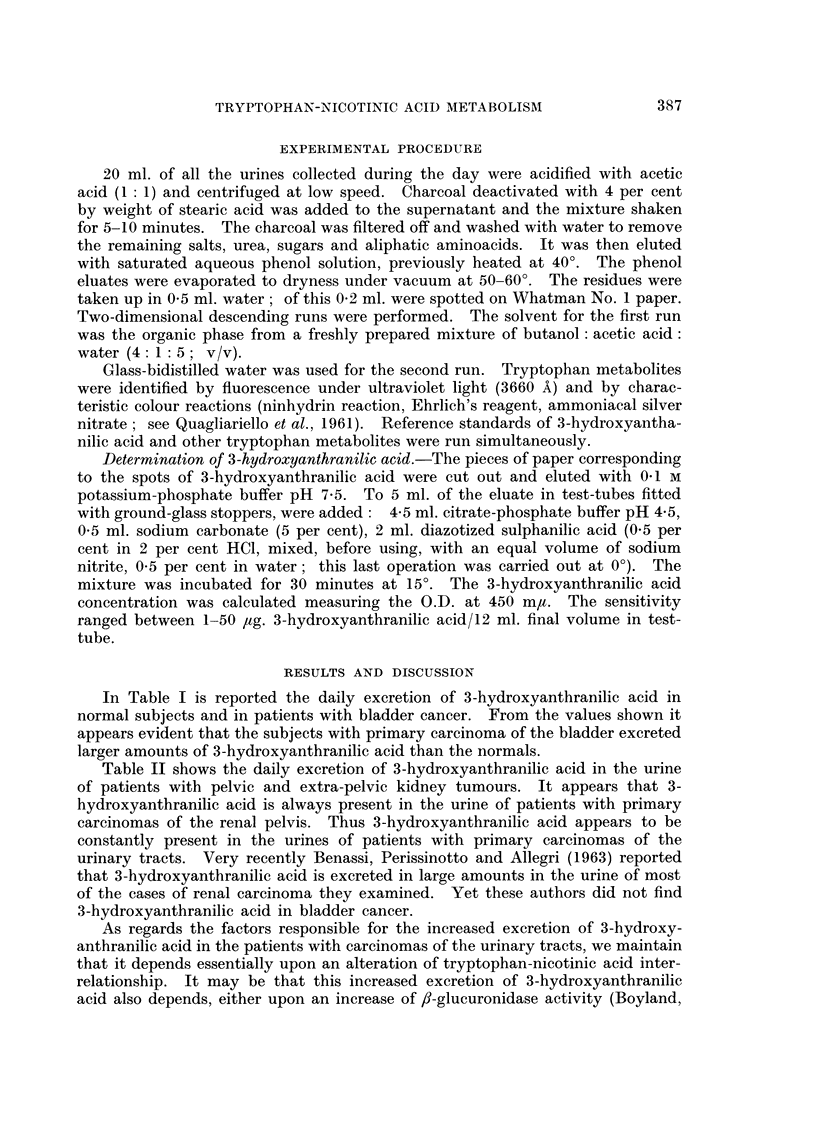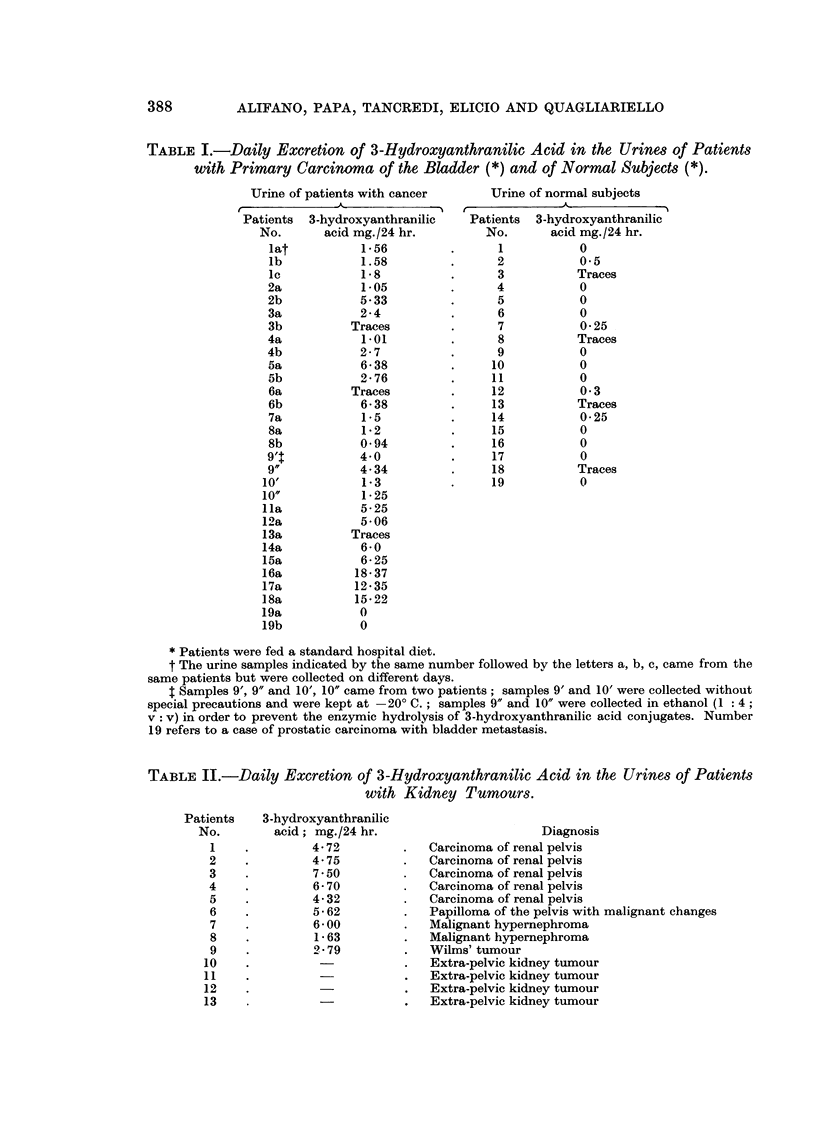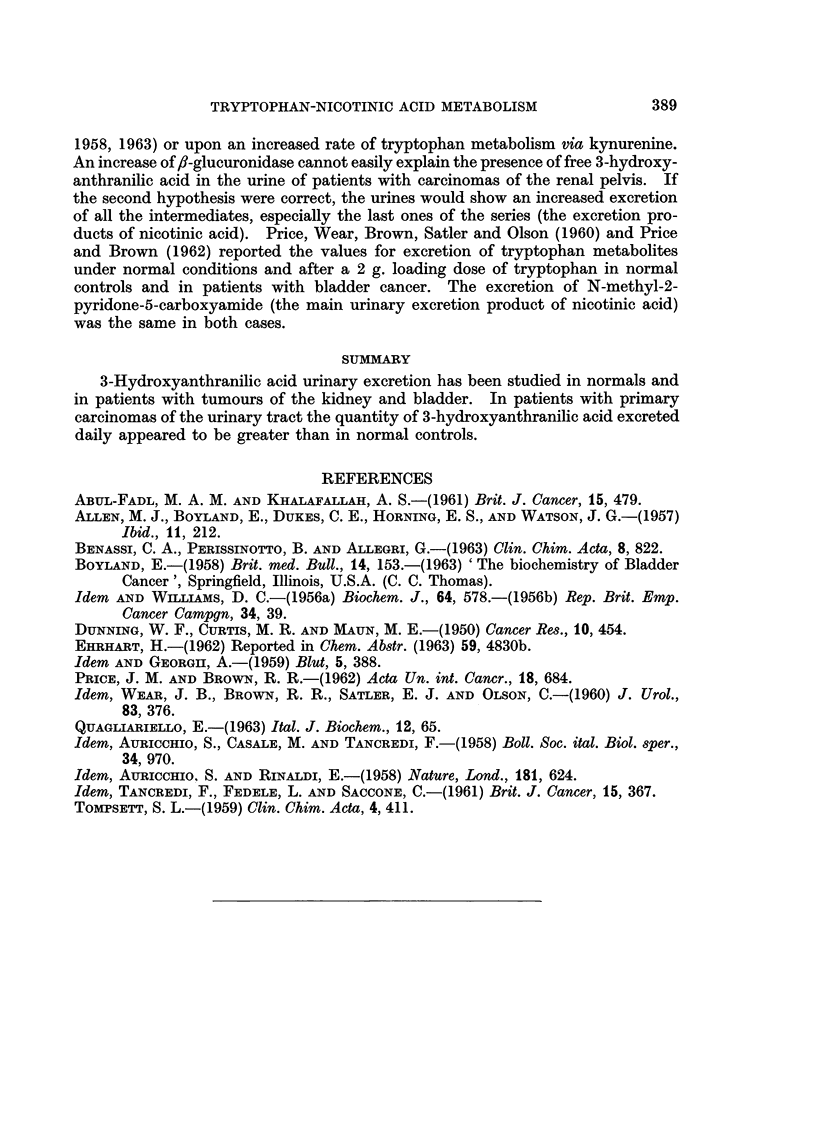# Tryptophan-Nicotinic Acid Metabolism in Patients with Tumours of the Bladder and Kidney

**DOI:** 10.1038/bjc.1964.44

**Published:** 1964-06

**Authors:** A. Alifano, S. Papa, F. Tancredi, M. A. Elicio, E. Quagliariello


### TRYPTOPHAN-NICOTINIC ACID METABOLISM IN PATIENTS WITH TUMOURS OF THE BLADDER AND KIDNEY

A. ALIFANO, S. PAPA, F. TANCREDI, M. A. ELICIO AND E. QUAGLIARIELLO

*From the Institute of Biological Chemistry, University of Bari, and Institute of Biological Chemistry, University of Naples, Italy*

Received for publication April 11, 1964

THE metabolic mechanism by which tryptophan is converted into nicotinic acid appears to be altered in several human diseases. Yet it has not been possible to demonstrate specific excretory patterns for the various diseases. The excretory pattern in the urines of patients with cancer of the bladder is certainly the one which presents the most specific and relevant alterations (Quagliariello, 1963).

Several authors have shown the presence of 3-hydroxyanthranilic acid in the urine of patients with cancer of the bladder : Boyland and Williams using column (1956a) and paper chromatography (1956b) ; Quagliariello, Auricchio, Casale and Tancredi (1958) by means of paper chromatography ; Tompsett (1959) and Abul-Fadl and Khalafallah (1961) with a colorimetric method. Quagliariello, Tancredi, Fedele and Saccone (1961) found that the amounts of 3-hydroxyanthranilic acid, 3-hydroxynurenine and anthranilic acid excreted by bladder cancer patients were reduced by giving large doses of nicotinamide. Allen, Boyland, Dukes, Horning and Watson (1957) have shown that biological ortho-aminophenols, such as 3-hydroxyanthranilic acid and 3-hydroxykynurenine, can induce experimental cancer of the bladder. Dunning, Curtis and Maun (1950) reported that cancer of the urinary bladder, caused by 2-acetamido-fluorene, arose in practically all cases in rats fed on a diet containing a high quantity of tryptophan. Further, Quagliariello, Auricchio and Rinaldi (1958) found that on oral administration of 2-acetamido-fluorene, 3-hydroxyanthranilic acid ⟶ quinolinic acid conversion in rat liver was inhibited to a large extent. This metabolic block would be expected to cause accumulation of carcinogenic substances such as 3-hydroxyanthranilic acid and 3-hydroxynurenine ; this could give a tentative explanation of the above reported findings of Dunning *et al.* Recently Ehrhart and Georgii (1959) reported that 3-hydroxyanthranilic acid can induce myeloid forms of leukaemia in mice (RFH-strain) with a low incidence of spontaneous leukaemia. The leukaemia rate following the administration of 3-hydroxyanthranilic acid could be increased by blocking 3-hydroxyanthranilic acid oxidase with 2-acetamido-fluorene (Ehrhart, 1962).

These studies prompted our further researches on the eventual connection between tryptophan metabolism and carcinogenesis. In the present work the excretion of 3-hydroxyanthranilic acid in the urine of patients with tumours of the urinary bladder and of the kidney has been studied. None of the patients with bladder cancer was engaged in the handling of aromatic amines.

### EXPERIMENTAL PROCEDURE

20 ml. of all the urines collected during the day were acidified with acetic acid (1 : 1) and centrifuged at low speed. Charcoal deactivated with 4 per cent by weight of stearic acid was added to the supernatant and the mixture shaken for 5–10 minutes. The charcoal was filtered off and washed with water to remove the remaining salts, urea, sugars and aliphatic aminoacids. It was then eluted with saturated aqueous phenol solution, previously heated at 40°. The phenol eluates were evaporated to dryness under vacuum at 50–60°. The residues were taken up in 0·5 ml. water ; of this 0·2 ml. were spotted on Whatman No. 1 paper. Two-dimensional descending runs were performed. The solvent for the first run was the organic phase from a freshly prepared mixture of butanol : acetic acid : water (4 : 1 : 5 ; v/v).

Glass-bidistilled water was used for the second run. Tryptophan metabolites were identified by fluorescence under ultraviolet light (3660 Å) and by characteristic colour reactions (ninhydrin reaction, Ehrlich's reagent, ammoniacal silver nitrate ; see Quagliariello *et al.*, 1961). Reference standards of 3-hydroxyanthanilic acid and other tryptophan metabolites were run simultaneously.

*Determination of* 3-*hydroxyanthranilic acid.*—The pieces of paper corresponding to the spots of 3-hydroxyanthranilic acid were cut out and eluted with 0·1 M potassium-phosphate buffer pH 7·5. To 5 ml. of the eluate in test-tubes fitted with ground-glass stoppers, were added : 4·5 ml. citrate-phosphate buffer pH 4·5, 0·5 ml. sodium carbonate (5 per cent), 2 ml. diazotized sulphanilic acid (0·5 per cent in 2 per cent HCl, mixed, before using, with an equal volume of sodium nitrite, 0·5 per cent in water ; this last operation was carried out at 0°). The mixture was incubated for 30 minutes at 15°. The 3-hydroxyanthranilic acid concentration was calculated measuring the O.D. at 450 m$\mu$. The sensitivity ranged between 1–50 $\mu$g. 3-hydroxyanthranilic acid/12 ml. final volume in test-tube.

### RESULTS AND DISCUSSION

In Table I is reported the daily excretion of 3-hydroxyanthranilic acid in normal subjects and in patients with bladder cancer. From the values shown it appears evident that the subjects with primary carcinoma of the bladder excreted larger amounts of 3-hydroxyanthranilic acid than the normals.

Table II shows the daily excretion of 3-hydroxyanthranilic acid in the urine of patients with pelvic and extra-pelvic kidney tumours. It appears that 3-hydroxyanthranilic acid is always present in the urine of patients with primary carcinomas of the renal pelvis. Thus 3-hydroxyanthranilic acid appears to be constantly present in the urines of patients with primary carcinomas of the urinary tracts. Very recently Benassi, Perissinotto and Allegri (1963) reported that 3-hydroxyanthranilic acid is excreted in large amounts in the urine of most of the cases of renal carcinoma they examined. Yet these authors did not find 3-hydroxyanthranilic acid in bladder cancer.

As regards the factors responsible for the increased excretion of 3-hydroxyanthranilic acid in the patients with carcinomas of the urinary tracts, we maintain that it depends essentially upon an alteration of tryptophan-nicotinic acid interrelationship. It may be that this increased excretion of 3-hydroxyanthranilic acid also depends, either upon an increase of $\beta$-glucuronidase activity (Boyland,

TABLE I.—*Daily Excretion of 3-Hydroxyanthranilic Acid in the Urines of Patients with Primary Carcinoma of the Bladder (*) and of Normal Subjects (*).*

| Urine of patients with cancer | | Urine of normal subjects | |
|---|---|---|---|
| Patients No. | 3-hydroxyanthranilic acid mg./24 hr. | Patients No. | 3-hydroxyanthranilic acid mg./24 hr. |
| 1a† | 1·56 | 1 | 0 |
| 1b | 1.58 | 2 | 0·5 |
| 1c | 1·8 | 3 | Traces |
| 2a | 1·05 | 4 | 0 |
| 2b | 5·33 | 5 | 0 |
| 3a | 2·4 | 6 | 0 |
| 3b | Traces | 7 | 0·25 |
| 4a | 1·01 | 8 | Traces |
| 4b | 2·7 | 9 | 0 |
| 5a | 6·38 | 10 | 0 |
| 5b | 2·76 | 11 | 0 |
| 6a | Traces | 12 | 0·3 |
| 6b | 6·38 | 13 | Traces |
| 7a | 1·5 | 14 | 0·25 |
| 8a | 1·2 | 15 | 0 |
| 8b | 0·94 | 16 | 0 |
| 9′‡ | 4·0 | 17 | 0 |
| 9″ | 4·34 | 18 | Traces |
| 10′ | 1·3 | 19 | 0 |
| 10″ | 1·25 | | |
| 11a | 5·25 | | |
| 12a | 5·06 | | |
| 13a | Traces | | |
| 14a | 6·0 | | |
| 15a | 6·25 | | |
| 16a | 18·37 | | |
| 17a | 12·35 | | |
| 18a | 15·22 | | |
| 19a | 0 | | |
| 19b | 0 | | |

* Patients were fed a standard hospital diet.

† The urine samples indicated by the same number followed by the letters a, b, c, came from the same patients but were collected on different days.

‡ Samples 9′, 9″ and 10′, 10″ came from two patients ; samples 9′ and 10′ were collected without special precautions and were kept at −20° C. ; samples 9″ and 10″ were collected in ethanol (1 : 4 ; v : v) in order to prevent the enzymic hydrolysis of 3-hydroxyanthranilic acid conjugates. Number 19 refers to a case of prostatic carcinoma with bladder metastasis.

TABLE II.—*Daily Excretion of 3-Hydroxyanthranilic Acid in the Urines of Patients with Kidney Tumours.*

| Patients No. | 3-hydroxyanthranilic acid ; mg./24 hr. | Diagnosis |
|---|---|---|
| 1 | 4·72 | Carcinoma of renal pelvis |
| 2 | 4·75 | Carcinoma of renal pelvis |
| 3 | 7·50 | Carcinoma of renal pelvis |
| 4 | 6·70 | Carcinoma of renal pelvis |
| 5 | 4·32 | Carcinoma of renal pelvis |
| 6 | 5·62 | Papilloma of the pelvis with malignant changes |
| 7 | 6·00 | Malignant hypernephroma |
| 8 | 1·63 | Malignant hypernephroma |
| 9 | 2·79 | Wilms' tumour |
| 10 | — | Extra-pelvic kidney tumour |
| 11 | — | Extra-pelvic kidney tumour |
| 12 | — | Extra-pelvic kidney tumour |
| 13 | — | Extra-pelvic kidney tumour |

1958, 1963) or upon an increased rate of tryptophan metabolism *via* kynurenine. An increase of $\beta$-glucuronidase cannot easily explain the presence of free 3-hydroxy-anthranilic acid in the urine of patients with carcinomas of the renal pelvis. If the second hypothesis were correct, the urines would show an increased excretion of all the intermediates, especially the last ones of the series (the excretion products of nicotinic acid). Price, Wear, Brown, Satler and Olson (1960) and Price and Brown (1962) reported the values for excretion of tryptophan metabolites under normal conditions and after a 2 g. loading dose of tryptophan in normal controls and in patients with bladder cancer. The excretion of N-methyl-2-pyridone-5-carboxyamide (the main urinary excretion product of nicotinic acid) was the same in both cases.

### SUMMARY

3-Hydroxyanthranilic acid urinary excretion has been studied in normals and in patients with tumours of the kidney and bladder. In patients with primary carcinomas of the urinary tract the quantity of 3-hydroxyanthranilic acid excreted daily appeared to be greater than in normal controls.

### REFERENCES

ABUL-FADL, M. A. M. AND KHALAFALLAH, A. S.—(1961) *Brit. J. Cancer*, **15**, 479.
ALLEN, M. J., BOYLAND, E., DUKES, C. E., HORNING, E. S., AND WATSON, J. G.—(1957) *Ibid.*, **11**, 212.
BENASSI, C. A., PERISSINOTTO, B. AND ALLEGRI, G.—(1963) *Clin. Chim. Acta*, **8**, 822.
BOYLAND, E.—(1958) *Brit. med. Bull.*, **14**, 153.—(1963) ' The biochemistry of Bladder Cancer ', Springfield, Illinois, U.S.A. (C. C. Thomas).
*Idem* AND WILLIAMS, D. C.—(1956a) *Biochem. J.*, **64**, 578.—(1956b) *Rep. Brit. Emp. Cancer Campgn*, **34**, 39.
DUNNING, W. F., CURTIS, M. R. AND MAUN, M. E.—(1950) *Cancer Res.*, **10**, 454.
EHRHART, H.—(1962) Reported in *Chem. Abstr.* (1963) **59**, 4830b.
*Idem* AND GEORGII, A.—(1959) *Blut*, **5**, 388.
PRICE, J. M. AND BROWN, R. R.—(1962) *Acta Un. int. Cancr.*, **18**, 684.
*Idem*, WEAR, J. B., BROWN, R. R., SATLER, E. J. AND OLSON, C.—(1960) *J. Urol.*, **83**, 376.
QUAGLIARIELLO, E.—(1963) *Ital. J. Biochem.*, **12**, 65.
*Idem*, AURICCHIO, S., CASALE, M. AND TANCREDI, F.—(1958) *Boll. Soc. ital. Biol. sper.*, **34**, 970.
*Idem*, AURICCHIO, S. AND RINALDI, E.—(1958) *Nature, Lond.*, **181**, 624.
*Idem*, TANCREDI, F., FEDELE, L. AND SACCONE, C.—(1961) *Brit. J. Cancer*, **15**, 367.
TOMPSETT, S. L.—(1959) *Clin. Chim. Acta*, **4**, 411.